# Molecular Detection and Typing of Pathogenic *Leptospira* in Febrile Patients and Phylogenetic Comparison with *Leptospira* Detected among Animals in Tanzania

**DOI:** 10.4269/ajtmh.19-0703

**Published:** 2020-08-03

**Authors:** Kathryn J. Allan, Michael J. Maze, Renee L. Galloway, Matthew P. Rubach, Holly M. Biggs, Jo E. B. Halliday, Sarah Cleaveland, Wilbrod Saganda, Bingileki F. Lwezaula, Rudovick R. Kazwala, Blandina T. Mmbaga, Venance P. Maro, John A. Crump

**Affiliations:** 1 Boyd Orr Centre for Population and Ecosystem Health, Institute of Biodiversity, Animal Health and Comparative Medicine, University of Glasgow, Glasgow, United Kingdom;; 2 Centre for International Health, University of Otago, Dunedin, New Zealand;; 3 Department of Medicine, University of Otago, Christchurch, New Zealand;; 4 Bacterial Special Pathogens Branch, US Centers for Disease Control and Prevention, Atlanta, Georgia;; 5 Division of Infectious Diseases and International Health, Duke University Medical Center, Durham, North Carolina;; 6 Duke Global Health Institute, Duke University, Durham, North Carolina;; 7 Programme for Emerging Infectious Diseases, Duke-National University of Singapore Medical School, Singapore, Singapore;; 8 Mawenzi Regional Referral Hospital, Moshi, Tanzania;; 9 Department of Veterinary Medicine and Public Health, Sokoine University of Agriculture, Morogoro, Tanzania;; 10 Kilimanjaro Christian Medical Centre, Moshi, Tanzania;; 11 Kilimanjaro Christian Medical University College, Tumaini University, Moshi, Tanzania;; 12 Kilimanjaro Clinical Research Institute, Moshi, Tanzania

## Abstract

Molecular data are required to improve our understanding of the epidemiology of leptospirosis in Africa and to identify sources of human infection. We applied molecular methods to identify the infecting *Leptospira* species and genotypes among patients hospitalized with fever in Tanzania and compared these with *Leptospira* genotypes detected among animals in Tanzania to infer potential sources of human infection. We performed *lipL32* real-time PCR to detect the presence of pathogenic *Leptospira* in acute-phase plasma, serum, and urine samples obtained from study participants with serologically confirmed leptospirosis and participants who had died with febrile illness. *Leptospira* blood culture was also performed. In positive specimens, we performed species-specific PCR and compared participant *Leptospira secY* sequences with *Leptospira* reference sequences and sequences previously obtained from animals in Tanzania. We detected *Leptospira* DNA in four (3.6%) of 111 participant blood samples. We detected *Leptospira borgpetersenii* (one participant, 25.0%), *Leptospira interrogans* (one participant, 25.0%), and *Leptospira kirschneri* (one participant, 25.0%) (one [25%] undetermined). Phylogenetic comparison of *secY* sequence from the *L. borgpetersenii* and *L. kirschneri* genotypes detected from participants was closely related to but distinct from genotypes detected among local livestock species. Our results indicate that a diverse range of *Leptospira* species is causing human infection. Although our analysis suggests a close relationship between *Leptospira* genotypes found in people and livestock, continued efforts are needed to obtain more *Leptospira* genetic material from human leptospirosis cases to help prioritize *Leptospira* species and genotypes for control.

## INTRODUCTION

Leptospirosis is a zoonotic disease, with an estimated annual incidence of up to approximately 100 cases per 100,000 people in Tanzania.^1^ Previous studies in Tanzania have identified contact with cattle and working in rice fields as risk factors for acute human leptospirosis,^2,3^ but further data are needed to understand transmission pathways and to confirm sources of *Leptospira* infection for people. Pathogenic *Leptospira* have been detected by culture or nucleic acid amplification methods in a number of animal hosts in Tanzania including cattle, goats, sheep, and rodents.^4,5^ Sequence-based species identification of Tanzanian animal isolates and DNA indicates that *Leptospira borgpetersenii*, *Leptospira interrogans*, and *Leptospira kirschneri* are circulating in animal populations in Tanzania. However, the contributions, and relative importance, of these *Leptospira* species to human disease in Tanzania are unclear.

Direct detection of *Leptospira* by culture can be challenging in humans as *Leptospira* are fastidious organisms that may take weeks to grow in culture, limiting the utility of this approach to provide a timely diagnosis for an acutely unwell patient.^6^ Because of the challenges of *Leptospira* culture in vitro, molecular diagnostic assays based on nucleic acid amplification tests (NAAT) including real-time PCR of the *lipL32* gene have been developed to aid diagnosis. PCR techniques have also been used to directly determine the species of infecting *Leptospira* for some of the more common pathogenic *Leptospira* species*.* This has been achieved using PCR-based amplification of *secY* and *ompL1* genes using species-specific primers and probes to show variation between *Leptospira* species.^7^ The sensitivity of these assays has not been fully determined but when used in a two-step algorithm with a *lipL32* assay was able to identify four common pathogenic *Leptospira* species (*L. borgpetersenii*, *L. interrogans*, *L. kirschneri*, and *Leptospira noguchii*).^7^ Sequence-based typing schemes of selected gene targets (e.g., 16S rRNA *rrs*, *secY*, and *lfb1*)^8–10^ have also been developed for *Leptospira.* Amplification of a ∼435-bp fragment of the *sec*Y gene has been shown to have good phylogenetic discrimination between pathogenic *Leptospira* species and has been widely used in the East African region.^4,8,11^ Sequence-based approaches have not only been applied to *Leptospira* isolates^8^ but can also be applied directly to clinical samples to determine the infecting species and genotype, and to investigate links between human and animal *Leptospira* infection.^12^

In this study, we aimed to characterize the genetic diversity of human *Leptospira* infection in northern Tanzania and to infer possible sources of human infection. We applied direct pathogen detection methods including culture and NAAT to detect *Leptospira* infection and determine the infecting *Leptospira* species among febrile patients in northern Tanzania. We then used sequence-based analysis of the *secY* gene to compare pathogenic *Leptospira* genotypes detected in patients with those previously reported in animals in Tanzania to advance our understanding of the relationship between human and animal *Leptospira* infection.

## METHODS

### Study setting.

Our study was performed among patients presenting to the Kilimanjaro Christian Medical Centre (KCMC), a 450-bed zonal referral hospital, and to Mawenzi Regional Referral Hospital (MRRH), a 300-bed regional referral hospital. Both hospitals are located in the Moshi municipal district in northern Tanzania. Moshi (population ∼180,000) is the administrative capital of the Kilimanjaro Region (population ∼1.6 million), which is located in the north of Tanzania.

### Study procedures and participants.

To detect the presence of *Leptospira* among febrile patients, we used plasma, serum, and urine collected from patients enrolled in two fever etiology studies that have been previously described.^2,13^ Briefly, we enrolled adult and pediatric inpatients admitted with a fever at the KCMC and MRRH from September 2007 through August 2008 (study 1) and from February 2012 through May 2014 (study 2). A questionnaire was administered to each participant that included questions on the duration of illness and any antibacterial treatment received before presentation at the hospital. We used participant questionnaire responses to estimate the duration of their fever (median and range) before sample collection.

Study personnel drew blood from each participant for serology, and participants were asked to return 4–6 weeks after enrollment for collection of a convalescent serum sample (both studies). We also collected acute urine samples from participants in study 1 and acute blood samples for *Leptospira* culture from participants enrolled in study 2.

### *Leptospira* culture.

For *Leptospira* culture, three drops of whole blood were inoculated immediately into 6-mL Ellinghausen–McCullough–Johnson–Harris culture media supplemented with 5-fluorouracil. Inoculated culture media were batch-shipped to the U.S. CDC where they were kept at 30°C for up to 6 months and monitored weekly for *Leptospira* growth by darkfield microscopic examination.

### Selection of participants for real-time PCR.

We selected participants for real-time PCR testing based on predetermined serological case definitions. In both studies, microscopic agglutination testing (MAT) was performed on acute and convalescent serum samples using a panel of 20 serovars belonging to 17 serogroups following standard laboratory procedures as previously described (Supplemental Table).^13–15^ Participants who had either a ≥ 4-fold rise in MAT titers between acute and convalescent serum samples or a single reciprocal MAT titer ≥ 800 to any serovar on acute or convalescent *Leptospira* were considered to be positive for acute leptospirosis and were selected for real-time PCR.^16^ The predominant reactive serogroup for each was defined as the serogroup containing the serovar with the highest agglutinating titer. In addition, we also selected study participants who had died before study follow-up for inclusion in our study (subsequently referred to as decedent participants). Such participants had provided only an acute-phase serologic sample, and as MAT is considered insensitive during the acute phase of illness, we included these samples to allow for missed cases within the decedent participant group.^17^

### Molecular detection of *Leptospira* DNA.

We performed real-time PCR testing to detect pathogenic *Leptospira* DNA in archived acute plasma and urine samples for participants recruited from study 1, and in acute serum samples for participants recruited from study 2. First, we extracted DNA from plasma and serum using the QIAamp^®^ DNA Blood Mini Kit (Qiagen, Hilden, Germany) according to the manufacturer’s instructions with a final elution volume of 100 μL to increase DNA concentration. DNA was extracted from urine using QIAamp DNA Blood Mini Kit with a pretreatment step designed to maximize the yield of bacterial DNA from biological samples.^18–20^ Briefly, up to 1,000 μL of urine was centrifuged at 10,000 × *g* for 10 minutes. The resulting pellet was washed in 200 μL Tris-EDTA (TE) buffer (Biotechnology grade, pH 8.0; VWR International Ltd., Lutterworth, United Kingdom) and then resuspended in an enzyme mix containing 50 μL lysozyme (10 mg/mL), 50 μL mutanolysin (1 mg/mL), and 4 μL lysostaphin (1 mg/mL) made up to a final volume of 200 μL with TE buffer, and incubated at 37°C for 1 hour. Subsequently, 180 μL lysis buffer (Buffer AL, Qiagen) and 20 μL proteinase K were added to each sample, and extraction was completed following the standard spin column extraction protocol.

To detect pathogenic *Leptospira* DNA in participant samples, we used a real-time PCR assay targeting the *lipL32* gene following previously published protocols,^21,22^ using the ABI 7500 real-time PCR system (Thermo Fisher Scientific, Waltham, MA). Assays were performed at National Collaborating Centre for Reference and Research on Leptospirosis, Dutch Royal Tropical Institute (now Amsterdam Medical Centre), Amsterdam, the Netherlands (Study 1), and at the Bacterial Special Pathogens Laboratory, U.S. CDC, Atlanta, GA (study 2). Primers and probes are shown in Table 1. Inhibition was evaluated using an endogenous internal positive control (human *rnaseP*) (study 1) or an exogenous positive control (Applied Biosystems). Each real-time reaction run included DNA extracted from a pure culture of *L. interrogans* as a positive control (study 1: *L. interrogans* serovar Copenhagenii strain Wijnberg supplied by the National Leptospirosis Reference Laboratory, Amsterdam, the Netherlands; study 2: *L. interrogans* serovar Icterohaemorrhagiae strain RGA from the American Type Culture Collection [ATCC] number 43642).^24^ In study 1, the master mix contained (per reaction) 1.25 μL of each primer, used at a concentration of 10 μM; 0.25 μL probe (10 μM concentration); 0.5 μL of ROX (1:10 dilution); 4.25 μL nuclease-free water; 12.5 μL Platinum quantitative PCR (qPCR) Supermix-UDG (Thermo Fisher Scientific, Waltham, MA); and 5 μL of the sample. Inhibition was evaluated in a separate qPCR reaction run on the same plate. The master mix for this reaction contained (per reaction) 1 μL of each primer (10 μM concentration), 0.3 μL probe (10 μM concentration), 0.5 μL ROX (1:10 dilution), 4.7 μL nuclease-free water, 12.5 μL Platinum qPCR Supermix-UDG (Thermo Fisher Scientific), and 5 μL of the sample. For study 2, the master mix contained (per reaction) 0.9 μL of each primer (20 μM concentration), 0.5 μL probe (5 μM concentration), 0.3 μL nuclease-free water, 10 μL PerfeCTa^®^ qPCR ToughMix^®^, Low ROX^™^ (Quanta Biosciences, Gaithersburg, MD), 2 μL 10X Exo IPC Mix, 0.4 μL 50X Exo IPC DNA, and 5 μL of the sample. For both studies, the amplification protocol consisted of 2 minutes at 50°C and 10 minutes at 95°C, followed by 45 cycles of amplification (95°C for 15 seconds and 60°C for 60 seconds [study 1] or 58°C for 60 seconds [study 2]). PCR-grade water was used as a non-template, negative control, and included in duplicate in each PCR run. Samples were tested in duplicate. Reaction runs were considered valid when both replicates of the *L. interrogans* control amplified with cycle threshold (Ct) values < 40 and when all replicates of the negative controls showed no evidence of amplification. Samples with a Ct value < 40 were considered positive for pathogenic *Leptospira* infection.

**Table 1:** Real-time PCR methods used to target genes of pathogenic *Leptospira* spp. among patients hospitalized with fever, Tanzania, 2007–2008 and 2012–2014

Genus/species	Target gene	Primer/probe name	Sequence (5′-3′)	Annealing temp (°C)	Cycles
Pathogenic *Leptospira* spp.	*lipL32*^22^	LipL32-45F	AAG CAT TAC CGC TTG TGG TG	60	40
		LipL32-286R	GAA CTC CCA TTT CAG CGA TT		
		LipL32-189P	FAM-AA AGC CAG GAC AAG CGC CG-BHQ1		
*Leptospira borgpetersenii*	*ompL1*^7^	F_bpn	GAT TCG GGT TAC AAT TAG ACC	65	45
		R_bpn1	TTG ATC TAA CCG GAC CAT AGT		
*Leptospira interrogans*	*secY*^7^	PFLint2	CTT GAG-CCT GCG CGT TAY C	63	45
		PRLint2	CCG ATA ATT CCA GCG AAG ATC		
		TaqLint2	TET-CTC ATT TGG TTA GGA GAA CAG ATC A-BHQ1		
*Leptospira kirschneri*	*secY*^7^	F_nery	CTG GCT TAA TCA ATG CTT CTG	60	45
		R_nery	CTC TTT CGG TGA TCT GTT CC		
		TqM_nery	Texas Red-CAG TTC CAG TTG TAA TAG ATA AGA TTC-BHQ2		
*Leptospira noguchii*	*secY*^7^	FLnog2	TCA GGG TGT AAG AAA GGT TC	63	45
		RLnog2	CAA AAT TAA AGA AGA AGC AAA GAT		
		TaqLnog	FAM-CGA TTG GCT TTT TGC TTG AAC CAT C-BHQ1		
		TqM_bpn	Cy5.5 (Quasar 705)-TAC TAA GGA TGG TTT GGA CGC TGC-BHQ2		
Pan *Leptospira* spp.	*SecY*^23^	SecYFd	5′-ATG CCG ATC ATY TTY GCT TC-3′	52°C	45
		secYR3	5′-TTC ATG AAG CCT TCA TAA TTT CTC A-3′		

### Identification of infecting *Leptospira* species and genotype.

For samples that were positive for pathogenic *Leptospira* DNA by *lipL32* real-time PCR assay, we performed real-time PCR using sets of *Leptospira* species–specific probes and flanking primers designed for the direct detection of common *Leptospira* species in clinical samples as shown in Table 1. Primers and probes targeting the *secY* gene were used for the detection of *L. interrogans*, *L. kirschneri*, and *Leptospira noguchi.* Primers and probes targeting the *ompL1* gene were used for the detection of *L. borgpetersenii.*^7^ In each run, we included negative controls and species-specific positive controls (*L. borgpetersenii* strain Ballum Mus 127 DMSO 4/21/15; *L. interrogans* strain Icterohaemorrhagiae RGA, ATCC 43462; *L. kirschneri* serovar Cynopteri strain 3522C, ATCC 49945; and *L. noguchi* strain 2001034031).

For samples that were positive by *lipL32* real-time PCR, we also amplified and sequenced a ∼435-base pair (bp) fragment of the *sec*Y gene to investigate the infecting genotype within each *Leptospira* species. PCR amplification was performed using previously published protocols. PCR amplicons were purified using the QIAquick PCR Purification Kit (Qiagen), and Sanger sequencing was performed by Source Bioscience Ltd. (Nottingham, United Kingdom).

### Phylogenetic analysis of the *secY* gene.

We performed phylogenetic analysis of the *secY* gene using MEGA7.0 software.^25^ Final sequences were aligned and compared with sequences from reference serovars accessed through GenBank^8,26^ and with sequences from other studies in Tanzania^4,27^ using the ClustalW algorithm. Notably, included in the comparison were sequences of *Leptospira* detected in Tanzanian livestock in the Moshi municipal district during the same time period as study 2 (2013–2014). This included 17 *Leptospira* sequences derived from cattle samples, one sequence derived from a goat sample, and one derived from a sheep sample.^4^ We also included published sequences from *Leptospira* reference serovars,^8^ and from serovars previously isolated in Tanzania in our final alignment.^27^ The model test function in MEGA7.0 was used to select the most appropriate nucleotide substitution model for the aligned sequences. The final phylogenetic tree was generated using a maximum likelihood method with 500 bootstraps repeats as a test of relationships between the aligned sequences.

### Research ethics.

This study was conducted in accordance with the World Medical Association Declaration of Helsinki. It was approved by the KCMC Research Ethics Committee (#295), the Tanzania National Institutes for Medical Research National Ethics Coordinating Committee (NIMR/HQ/R.8cNo1. 11/283 and NIMR/HQ/R.8a/Vol.IX/1499), Duke Health Institutional Review Board (IRB#Pro00016134), the University of Otago Human Ethics Committee (Health) (H15/055), and the University of Glasgow College of Medical, Veterinary, and Life Sciences Ethics Committee (Project No. 200120020). Written informed consent was obtained from all participants. Study data are available at http://dx.doi.org/10.5525/gla.researchdata.881.

## RESULTS

In total, 1,849 participants who were recruited into the two fever etiology studies had blood drawn for leptospirosis serology, and 1,294 (70.0%) had *Leptospira* blood culture. MAT serological testing for leptospirosis was performed on paired acute and convalescent samples for 1,225 (66.3%) participants, and there were 108 decedents. A total of 109 (5.9%) participants met our selection criteria for real-time PCR testing for *Leptospira* and had samples available for testing*.* These included samples from 81 participants who met our serologic case definition of leptospirosis and 28 decedent participants. Of 109 participants, 106 (98.1%) participants had blood derivatives, including plasma in 58 (53.2%) and serum in 48 (44.0%) available for testing, and 30 (27.5%) had urine available for testing.

Data on fever duration and self-reported antibacterial use were available from 108 (99.1%) participants undergoing PCR testing. The median (range) reported duration of the fever before sample collection was 7 (1–366) days. Of participants, 64 (58.7%) reported antibacterial use before enrollment.

### Pathogenic *Leptospira* testing and speciation.

*Leptospira* culture was negative on all 1,294 participants. By real-time PCR, we detected pathogenic *Leptospira* DNA in three (3.7%) of 81 participants with serologically confirmed acute leptospirosis and one (3.6%) of 28 decedent participants (Table 2). The decedent participant did not meet our serologic case definition of leptospirosis (low titer in the acute sample and no convalescent sample available). All positive PCR reactions were from serum samples. Of four real-time PCR-positive samples, species-specific real-time PCR identified one (25%) as positive for *L. borgpetersenii*, one (25%) *L. interrogans*, and one (25%) *L. kirschneri*, each in a single study participant (Table 2). In the single decedent sample (25%) that was *lipL32* real-time PCR positive, we were unable to amplify DNA using the species-specific real-time PCR reactions.

**Table 2:** *LipL32* and species-specific RT-PCR CT values, and MAT predominant serogroup in participants with *Leptospira* DNA detected, Tanzania, 2012–2014

Participant number	1	2	3	4
PCR				
*LipL32* CT value	38.7	36.3	32.3	38.4
*L. borgpetersenii* CT value	ND	37.8	ND	ND
*Leptospira interrogans* CT value	37.4	ND	ND	ND
*L. kirschneri* CT value	ND	ND	37.8	ND
*Leptospira noguchii* CT value	ND	ND	ND	ND
*Leptospira* species (*secY* sequencing)	NA	*L. borgpetersenii*	*L. kirschneri*	NA
MAT				
Predominant MAT serogroup	Sejroe	Pyrogenes	Sejroe	Pyrogenes
Acute reciprocal titer	0	0	0	400
Convalescent reciprocal titer	200	800	12,800	NA

CT = cycle threshold; *L. borgpetersenii* = *Leptospira borgpetersenii*; *L. kirschneri = Leptospira kirschneri*; MAT = microscopic agglutination testing; NA = not available; ND = not detected.

### Comparison between genetic and serological results.

When compared with the MAT serological results, all three participants with real-time PCR-positive samples met the case definition for acute leptospirosis by virtue of a 4-fold rise in MAT titer between the acute and convalescent samples. The predominant reactive serogroup was Sejroe for the participants with *L. interrogans* and *L. kirschneri* infection, and was Pyrogenes for the participant with *L. borgpetersenii* infection (Table 2). For the real-time PCR-positive decedent, a single titer of 1:400 against serogroup Pyrogenes was obtained by MAT.

### *Leptospira* phylogenetic analysis.

**Figure 1.**
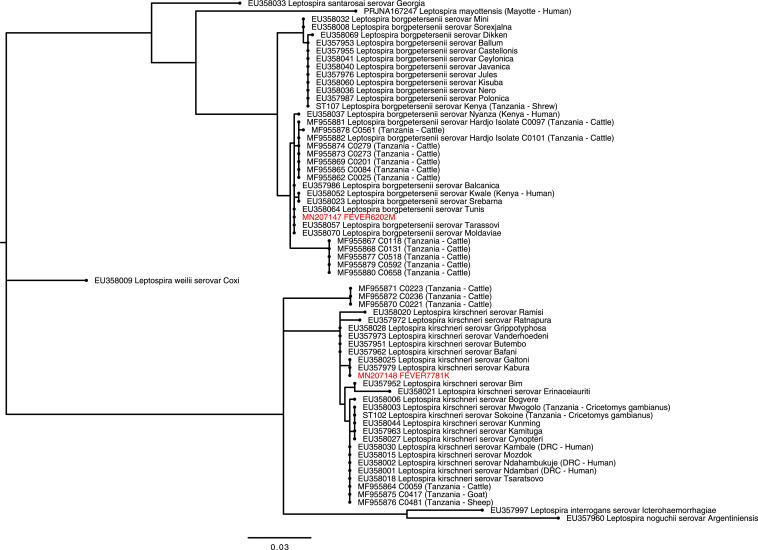
Phylogenetic tree showing the relationship between *Leptospira secY* gene (434-bp fragment) derived from human infections in Tanzania with sequence from reference serovars^8^ and previously published sequences from human and animal infection in Tanzania^4,27^ The phylogenetic tree was constructed using the maximum likelihood method using the Tamura 3-parameter (T92) nucleotide substitution model with a discrete gamma distribution.^44^ The tree with the highest log likelihood is shown. Sequences from our study are labeled with unique identifiers (FEVERXXXX) and GenBank accession numbers and highlighted in red. Sequences generated from reference *Leptospira* serovars and from other studies in Tanzania are shown and labeled with GenBank accession numbers and ST types, respectively.^45^ Expanded clades show reference serovars most closely related to the study genotypes. Clades of more distantly related species are collapsed and labeled with species names only. Country locations and host are shown in parenthesis for East African studies. DRC = Democratic Republic of Congo; sv = serovar; ST = sequence types. This figure appears in color at www.ajtmh.org.

*SecY* sequences were obtained from two (50.0%) of four participants whose samples were positive by *lipL32* real-time PCR. Comparison with published *secY* reference sequences^8^ confirmed the infecting *Leptospira* species as *L. borgpetersenii* and *L. kirschneri* (Figure 1, Table 2). When compared with reference serovars, *L. borgpetersenii secY* sequence from a participant in our study was 100% identical to reference sequences from *L. borgpetersenii* serovar Balcanica (EU357986), Moldaviae (EU358070), Tarassovi (EU358057), and Tunis (EU358064). *L. kirschneri secY* sequence generated in our study was 100% identical to sequences obtained from reference serovars including *L. kirschneri* serovar Galtoni (EU358025), and serovar Kabura (EU 357979).

Sequences obtained from participants in our study showed not only a high degree of similarity but also some minor distinctions when compared with *Leptospira secY* sequences obtained from animals in Tanzania (Figure 1).^4,27^ *Leptospira borgpetersenii* sequences were highly similar (99.8% of 434 bp) to sequences of *L. borgpetersenii* serovar Hardjo (Hardjo-bovis) isolated from cattle in the Moshi municipal district, but with a single nucleotide substitution in the human-derived sequence. *L. kirschneri* sequences were also highly similar (99.1% of 434 bp) but differed by four nucleotide substitutions from genotypes detected in cattle, sheep, and goats sampled in the Moshi municipal district.

## DISCUSSION

We detected *Leptospira* DNA in four participants with acute febrile illness in northern Tanzania, including one patient who died with acute leptospirosis. Despite the small number of real-time PCR-positive samples, our results show a striking amount of diversity in infecting *Leptospira* species. Three *Leptospira* species—*L. borgpetersenii*, *L. interrogans*, and *L. kirschneri—*were detected in participants with acute febrile illness. Sequence-based analysis showed a high degree of similarity between *secY* sequence obtained from *L. borgpetersenii* and *L. kirschneri* genotypes infecting people and *L. borgpetersenii* and *L. kirschneri* genotypes detected in local cattle, goats, and sheep also sampled in the Moshi municipal district. The findings of our study corroborate previous serological findings that detected multiple *Leptospira* types are circulating in Tanzania and support the hypothesis that livestock are a potential source of human infection.^2,4,13^

To date, little information has been available regarding the predominant *Leptospira* species implicated in human disease in Tanzania.^28^ PerfeCTa qPCR ToughMix, Low ROX *interrogans*, and *L. kirschneri* have previously been implicated as a cause of human infection elsewhere in Tanzania although the methods used in the earlier study lacked sufficient resolution to discriminate to the species level.^27^ Our approach, which used a combination of species-specific PCR and sequence-based typing, has generated to our knowledge the first species-level identification of pathogenic *Leptospira* associated with human disease and provides robust evidence that multiple *Leptospira* types are contributing to human leptospirosis in Tanzania.

Phylogenetic analysis of a partial fragment of the *secY* gene enabled comparison with *L. borgpetersenii* and *L. kirschneri* genotypes identified in local livestock species. Sequences of the *Leptospira secY* gene obtained from our study participants showed a high degree of similarity to *secY* sequences obtained previously from livestock in Tanzania. In particular, *L. borgpetersenii* is commonly carried by cattle in northern Tanzania,^4^ and the genotype derived from a patient in our study was highly similar to that of *L. borgpetersenii* sequences obtained from cattle in Tanzania that were sampled during the same time period and same geographic region.^4^ However, a high degree of similarity was also seen in sequences from three *Leptospira* reference serovars including *L. borgpetersenii* serovar Balcanica, previously detected in cattle in Zimbabwe^29^; *L. borgpetersenii* serovar Tunis, previously detected from pigs in Tunisia^30^; and *L. borgpetersenii* serovar Tarassovi, which has been detected among a wide variety of wildlife and livestock from a broad geographical distribution.^31–36^ Likewise, the *L. kirschneri* genotype derived from a human participant in our study was similar to but distinct from sequences derived from livestock in Tanzania.^4^ This finding suggests a less recent common ancestor between the *L. kirschneri* genotypes detected in our study and those previously detected in livestock than for *L. borgpetersenii* genotypes.

Source attribution for *Leptospira* infection requires a thorough characterization of local *Leptospira* serovars or sequence types as well as a good understanding of the animal host range and potential transmission pathways for each of these types.^37,38^ The relatively small amount of published *Leptospira* sequence data from Tanzania, combined with the limited amount of typing that can be performed for *Leptospira* in the absence of cultured isolates, means that we are unable to robustly infer the sources of infection for the febrile patients enrolled in our study. However, based on our current understanding of the epidemiology and biology of these *Leptospira* species, we think that it is plausible that the three different *Leptospira* spp. detected in patients in our study have distinct epidemiology and transmission pathways. First, the three different *Leptospira* spp. detected in our study have previously been identified in different animal hosts in Tanzania. To date, *L. borgpetersenii* has only been detected in cattle and rodents in Tanzania, whereas *L. kirschneri* appears to have a broader animal host range including cattle, sheep, goats, and rodents.^4,27^ In addition, other studies have demonstrated biological differences between *Leptospira* species. For example, *L. borgpetersenii* has reduced environmental survival compared with *L. interrogans,* and hence, relies more heavily on direct contact for host-to-host transmission.^39^ Therefore, we hypothesize that infection transmission may be occurring under different environmental conditions for each of the *Leptospira* species identified, with important implications for public health strategies to reduce the burden of infection.

Overall, our study was limited by the small number of PCR-positive samples from human participants available for analysis. Our PCR approach had relatively low sensitivity and detected fewer than 4% of acute leptospirosis cases, despite focusing our selection on acute blood and urine samples from participants that met international guidelines serological case definitions for acute leptospirosis.^16^ Testing the entire cohort with both MAT and PCR is likely to have detected additional cases as although MAT using paired serum is the reference standard for serological diagnosis for leptospirosis, it has imperfect sensitivity as some patients will not seroconvert.^17,40–42^ Resource limitations preclude testing all participants with both assays.

Many studies report that MAT testing of paired serum samples detected cases that were unable to be detected by PCR,^17,40–42^ but the proportion of serologically confirmed cases who tested positive by PCR was surprisingly low in our study. One possible explanation for the surprisingly low proportion positive by PCR was that at least half of participants in our studies reported a fever for greater than 7 days before sample collection. PCR has highest sensitivity during the bacteremic phase of leptospirosis during first 5–7 days of symptoms.^43^ The high reported prevalence of prior antimicrobial use may have further reduced the proportion positive by culture and PCR in our studies by sterilizing blood before enrollment preventing bacterial growth, or reducing leptospiral load below the lower limit of detection for the real-time PCR assay. In addition, we performed the PCR assays on serum and plasma, which may have lower sensitivity than whole blood.^21^ Despite this limitation, the small amount of molecular data that we obtained has helped to expand our understanding of the diversity of *Leptospira* present in northern Tanzania and also provided some intriguing insights into the relationship between infecting *Leptospira* species and serogroup in patients with acute febrile illness. For example, Sejroe was the predominant reactive serogroup in two patients who each were infected with different *Leptospira* species: *L. borgpetersenii* and *L. interrogans.* This suggests that molecular typing may reveal greater diversity than serology alone. Furthermore, the use of *Leptospira* real-time PCR allowed us to detect a fatal case of leptospirosis in our patient cohort that may otherwise have been undetected and demonstrates the potential value of PCR in understanding the prevalence of fatal leptospirosis.

Our study has provided some new insights into the complexity of *Leptospira* transmission pathways in Tanzania. Given the relatively small amount of data and the high incidence of human leptospirosis in Tanzania,^1,15^ we think that further work is needed to determine the diversity of *Leptospira* species and genotypes contributing to both human and animal infection, and to understand sources of infection and epidemiological transmission sources for human leptospirosis. This could in turn inform efforts to control and prevent human disease.

Based on our findings, we recommend the combined use of both serological and NAAT diagnostic approaches to diagnose and investigate the epidemiology of human leptospirosis. This is particularly important where delays in presentation to healthcare facilities are common, or when there is a high prevalence of prehospital antibacterial use. Additional efforts to obtain *Leptospira* genetic material from human leptospirosis cases are needed to help prioritize *Leptospira* species and genotypes for control and critical insights into the links between human and animal infection and help understand transmission of this complex but important pathogen.

## Supplemental table

Supplemental materials

## References

[1] Biggs HM Hertz JT Munishi OM Galloway RL Marks F Saganda W Maro VP Crump JA, 2013 Estimating leptospirosis incidence using hospital-based surveillance and a population-based health care utilization survey in Tanzania. PLoS Negl Trop Dis 7: e2589. 24340122 10.1371/journal.pntd.0002589 PMC3855074

[2] Maze MJ 2018 Risk factors for acute human leptospirosis in northern Tanzania. PLoS Negl Trop Dis 12: e0006372. 29879114 10.1371/journal.pntd.0006372 PMC5991637

[3] Schoonman L Swai ES, 2009 Risk factors associated with the seroprevalence of leptospirosis, amongst at-risk groups in and around Tanga city, Tanzania. Ann Trop Med Parasitol 103: 711–718. 20030995 10.1179/000349809X12554106963393

[4] Allan KJ 2018 Assessment of animal hosts of pathogenic *Leptospira* in northern Tanzania. PLoS Negl Trop Dis 12: e0006444. 29879104 10.1371/journal.pntd.0006444 PMC5991636

[5] Mgode GF Machang’u RS Goris MG Engelbert M Sondij S Hartskeerl RA, 2006 New *Leptospira* serovar sokoine of serogroup Icterohaemorrhagiae from cattle in Tanzania. Int J Syst Evol Microbiol 56: 593–597. 16514033 10.1099/ijs.0.63278-0

[6] Levett PN, 2001 Leptospirosis. Clin Microbiol Rev 14: 296–326. 11292640 10.1128/CMR.14.2.296-326.2001 PMC88975

[7] Ferreira AS Costa P Rocha T Amaro A Vieira ML Ahmed A Thompson G Hartskeerl RA Inacio J, 2014 Direct detection and differentiation of pathogenic *Leptospira* species using a multi-gene targeted real time PCR approach. PLoS One 9: e112312. 25398140 10.1371/journal.pone.0112312 PMC4232388

[8] Victoria B Ahmed A Zuerner RL Ahmed N Bulach DM Quinteiro J Hartskeerl RA, 2008 Conservation of the *S*10-*spc*-alpha locus within otherwise highly plastic genomes provides phylogenetic insight into the genus *Leptospira*. PLoS One 3: e2752. 18648538 10.1371/journal.pone.0002752 PMC2481283

[9] Morey RE Galloway RL Bragg SL Steigerwalt AG Mayer LW Levett PN, 2006 Species-specific identification of *Leptospiraceae* by *16S* rRNA gene sequencing. J Clin Microbiol 44: 3510–3516. 17021075 10.1128/JCM.00670-06 PMC1594759

[10] Merien F Portnoi D Bourhy P Charavay F Berlioz-Arthaud A Baranton G, 2005 A rapid and quantitative method for the detection of *Leptospira* species in human leptospirosis. FEMS Microbiol Lett 249: 139–147. 16006065 10.1016/j.femsle.2005.06.011

[11] Dietrich M Wilkinson DA Soarimalala V Goodman SM Dellagi K Tortosa P, 2014 Diversification of an emerging pathogen in a biodiversity hotspot: *Leptospira* in endemic small mammals of Madagascar. Mol Ecol 23: 2783–2796. 24784171 10.1111/mec.12777

[12] Hamond C Pestana CP Medeiros MA Lilenbaum W, 2016 Genotyping of *Leptospira* directly in urine samples of cattle demonstrates a diversity of species and strains in Brazil. Epidemiol Infect 144: 72–75. 26076668 10.1017/S0950268815001363 PMC9507312

[13] Biggs HM 2011 Leptospirosis among hospitalized febrile patients in northern Tanzania. Am J Trop Med Hyg 85: 275–281. 21813847 10.4269/ajtmh.2011.11-0176 PMC3144825

[14] Goris MG Hartskeerl RA, 2014 Leptospirosis serodiagnosis by the microscopic agglutination test. Curr Protoc Microbiol 32: Unit 12E 5. 24510846 10.1002/9780471729259.mc12e05s32

[15] Maze MJ 2016 Comparison of the estimated incidence of acute leptospirosis in the Kilimanjaro Region of Tanzania between 2007–08 and 2012–14. PLoS Negl Trop Dis 10: e0005165. 27911902 10.1371/journal.pntd.0005165 PMC5135036

[16] CDC, 2013 Leptospirosis (Leptospira interrogans) 2013 Case Definition. Available at: https://wwwn.cdc.gov/nndss/conditions/leptospirosis/case-definition/2013/. Accessed October 21, 2015.

[17] Limmathurotsakul D Turner EL Wuthiekanun V Thaipadungpanit J Suputtamongkol Y Chierakul W Smythe LD Day NP Cooper B Peacock SJ, 2012 Fool’s gold: why imperfect reference tests are undermining the evaluation of novel diagnostics: a reevaluation of 5 diagnostic tests for leptospirosis. Clin Infect Dis 55: 322–331. 22523263 10.1093/cid/cis403 PMC3393707

[18] Qiagen, 2006 DNeasy® Blood & Tissue Handbook. Hilden, Germany: Qiagen.

[19] Bag S 2016 An improved method for high quality metagenomics DNA extraction from human and environmental samples. Sci Rep 6: 26775. 27240745 10.1038/srep26775 PMC4886217

[20] Gill C van de Wijgert JHHM Blow F Darby AC, 2016 Evaluation of lysis methods for the extraction of bacterial DNA for analysis of the vaginal microbiota. PLoS One 11: e0163148. 27643503 10.1371/journal.pone.0163148 PMC5028042

[21] Galloway RL Hoffmaster AR, 2015 Optimization of *LipL32* PCR assay for increased sensitivity in diagnosing leptospirosis. Diagn Microbiol Infect Dis 82: 199–200. 25912810 10.1016/j.diagmicrobio.2015.03.024 PMC6452440

[22] Stoddard RA, 2013 Detection of pathogenic *Leptospira* spp. through real-time PCR (qPCR) targeting the *LipL32* gene. Methods Mol Biol 943: 257–266. 23104295 10.1007/978-1-60327-353-4_17

[23] Ahmed A Engelberts MF Boer KR Ahmed N Hartskeerl RA, 2009 Development and validation of a real-time PCR for detection of pathogenic *Leptospira* species in clinical materials. PLoS One 4: e7093. 19763264 10.1371/journal.pone.0007093 PMC2740861

[24] Tindall BJ, 2014 ATCC 43642 replaces ATCC 23581 as the type strain of *Leptospira interrogans* (Stimson 1907) Wenyon 1926. Opinion 91. Judicial Commission of the International Committee on Systematics of prokaryotes. Int J Syst Evol Microbiol 64: 3584–3585. 25288661 10.1099/ijs.0.069179-0

[25] Kumar S Stecher G Tamura K, 2015 MEGA7: molecular evolutionary genetics analysis version 7.0 for bigger datasets. Mol Biol Evol 33: 1870–1874. 10.1093/molbev/msw054 PMC8210823 27004904

[26] Benson DA Karsch-Mizrachi I Clark K Lipman DJ Ostell J Sayers EW, 2011 GenBank. Nucleic Acids Res 40: D48–D53. 22144687 10.1093/nar/gkr1202 PMC3245039

[27] Mgode GF Machang’u RS Mhamphi GG Katakweba A Mulungu LS Durnez L Leirs H Hartskeerl RA Belmain SR, 2015 *Leptospira* serovars for diagnosis of leptospirosis in humans and animals in Africa: common *Leptospira* isolates and reservoir hosts. PLoS Negl Trop Dis 9: e0004251. 26624890 10.1371/journal.pntd.0004251 PMC4666418

[28] Muller SK Assenga JA Matemba LE Misinzo G Kazwala RR, 2016 Human leptospirosis in Tanzania: sequencing and phylogenetic analysis confirm that pathogenic *Leptospira* species circulate among agro-pastoralists living in Katavi-Rukwa ecosystem. BMC Infect Dis 16: 273. 27287703 10.1186/s12879-016-1588-x PMC4902944

[29] Feresu SB Steigerwalt AG Brenner DJ, 1999 DNA relatedness of *Leptospira* strains isolated from beef cattle in Zimbabwe. Int J Syst Bacteriol 49: 1111–1117. 10425768 10.1099/00207713-49-3-1111

[30] Bakoss P Chadli A, 1965 The pig, reservoir of *Leptospira mitis* in Tunisia. Arch Inst Pasteur Tunis 42: 85–91.

[31] Maze MJ 2017 The Global Distribution and Host Range of Leptospira Species and Serovars. 10th International Leptospirosis Society Meeting. Palmerston North, New Zealand.

[32] Robinson AJ Ramadass P Lee A Marshall RB, 1982 Differentiation of subtypes within *Leptospira interrogans* serovars Hardjo, Balcanica and Tarassovi, by bacterial restriction-endonuclease DNA analysis (BRENDA). J Med Microbiol 15: 331–338. 6288951 10.1099/00222615-15-3-331

[33] Field PR Santiago A Chan SW Patel DB Dickeson D Mitchell JL Devine PL Murphy AM, 2002 Evaluation of a novel commercial enzyme-linked immunosorbent assay detecting *Coxiella burnetii*-specific immunoglobulin G for Q fever prevaccination screening and diagnosis. J Clin Microbiol 40: 3526–3529. 12202611 10.1128/JCM.40.9.3526-3529.2002 PMC130813

[34] Carmona-Gasca CA Lara LL Castillo-Sanchez LO Ramirez-Ortega JM Ko A Palomera CL de la Pena-Moctezuma A, 2011 Detection of *Leptospira santarosai* and *L. kirschneri* in cattle: new isolates with potential impact in bovine production and public health. Veterinaria Mexico 42: 277–288.

[35] Aragon PR Jacalne AV Famatiga EG, 1965 Isolation of *Leptospira* from rats, dogs and pigs. Philippine J Sci 94: 45–54.

[36] Chernukha YG Isayeva RA Mustafayeva NI, 1969 Antigenic properties of some strains of *Leptospirae* of the Tarassovi serological group. Systematic positiion of the strain Perepelicin and new serological type Vietnam. J Hyg Epidemiol Microbiol Immunol (Prague) 13: 118–125. 5814135

[37] Guernier V Allan KJ Goarant C, 2017 Advances and challenges in barcoding pathogenic and environmental *Leptospira*. Parasitology 145: 595–607. 28716157 10.1017/S0031182017001147 PMC6010154

[38] Mather AE Vaughan TG French NP, 2015 Molecular approaches to understanding transmission and source attribution in nontyphoidal *Salmonella* and their application in Africa. Clin Infect Dis 61 (Suppl 4): S259–S265. 26449940 10.1093/cid/civ727

[39] Bulach DM 2006 Genome reduction in *Leptospira borgpetersenii* reflects limited transmission potential. Proc Natl Acad Sci USA 103: 14560–14565. 16973745 10.1073/pnas.0603979103 PMC1599999

[40] Schreier S Doungchawee G Chadsuthi S Triampo D Triampo W, 2013 Leptospirosis: current situation and trends of specific laboratory tests. Expert Rev Clin Immunol 9: 263–280. 23445200 10.1586/eci.12.110

[41] Agampodi SB Matthias MA Moreno AC Vinetz JM, 2012 Utility of quantitative polymerase chain reaction in leptospirosis diagnosis: association of level of leptospiremia and clinical manifestations in Sri Lanka. Clin Infect Dis 54: 1249–1255. 22354922 10.1093/cid/cis035 PMC3404689

[42] Thaipadungpanit J 2011 Diagnostic accuracy of real-time PCR assays targeting *16S* rRNA and *LipL32* genes for human leptospirosis in Thailand: a case-control study. PLoS One 6: e16236. 21283633 10.1371/journal.pone.0016236 PMC3026019

[43] Haake DA Levett PN, 2015 Leptospirosis in humans. Curr Top Microbiol Immunol 387: 65–97. 25388133 10.1007/978-3-662-45059-8_5 PMC4442676

[44] Tamura K, 1992 Estimation of the number of nucleotide substitutions when there are strong transition-transversion and G+C-content biases. Mol Biol Evol 9: 678–687. 1630306 10.1093/oxfordjournals.molbev.a040752

[45] Jolley KA Maiden MC, 2016 Leptospira spp. MLST Databases. Available at: http://pubmlst/org/leptospira. Accessed February 6, 2019.

