# Supplementary material for: Molecular Detection and Typing of Pathogenic Leptospira in Febrile Patients and Phylogenetic Comparison with Leptospira Detected among Animals in Tanzania
Source: Am J Trop Med Hyg. 2020 Aug 3;103(4):1427–34. doi: 10.4269/ajtmh.19-0703 (PMC7543812; doi:10.4269/ajtmh.19-0703)
Supplement: Supplementary file 1 [file tpmd190703.SD1.pdf]

Supplemental Table: Number of patients and samples (types) available for *lipL32* quantitative PCR testing for pathogenic *Leptospira* infection among patients hospitalized with fever, Tanzania, 2007-08 and 2012-14

Patient group	Number of patients	Number of serum samples (Positive/total tested)	Number of plasma samples (Positive/total tested)	Number of urine samples (Positive/total tested)
**Study 1: Cases**	43	-	0/40	0/25
**Study 1: Decedents**	18	-	0/18	0/5
**Study 2: Cases**	24	3/24	-	-
**Study 2 Decedents**	26	1/26	-	-
**Total**	**111**	**4/50**	**0/58**	**0/30**
